# Corrosion Behavior of High-Nitrogen Steel Hybrid Welded Joints Fabricated by Hybrid Laser–Arc Welding

**DOI:** 10.3390/ma16103617

**Published:** 2023-05-09

**Authors:** Xianxian You, Kai Ning, Di Bai, Yanwei Liu, Hong Zhang, Fengde Liu

**Affiliations:** 1 College of Mechanical and Electric Engineering, Changchun University of Science and Technology, Changchun 130022, China; 2 National Base of International Science and Technology Cooperation in Optics, Changchun 130022, China; 3 Changchun Hexin Machinery Manufacturing Co., Ltd., Changchun 130031, China

**Keywords:** high-nitrogen steel, laser–arc hybrid welding, heat input, corrosion behavior

## Abstract

To study the corrosion mechanism of high-nitrogen steel welds, this study investigated the effects of laser outputs on the corrosion behavior of high-nitrogen steel hybrid welded joints in hybrid laser–arc welding. The relationship between the ferrite content and laser output was characterized. The ferrite content increased with the increase in the laser power. The corrosion phenomenon first occurred at the two-phase interface, thereby forming corrosion pits. Ferritic dendrites were first corroded to form dendritic corrosion channels. Furthermore, first-principles calculations were performed to investigate the properties of the austenite and ferrite content. The work function and surface energy indicated that solid-solution nitrogen austenite exhibits a higher surface structural stability than austenite and ferrite. This study provides useful information for high-nitrogen steel weld corrosion.

## 1. Introduction

High-nitrogen austenitic stainless steels, also known as high-nitrogen steels, are produced through the process of austenitizing. This is achieved by replacing nickel with nitrogen during steelmaking. The mass fraction of nitrogen in austenitic steel exceeds 0.4%. Such steels are widely used in military equipment, medical devices, marine shipbuilding, and other fields, owing to their excellent mechanical properties and corrosion resistance [1,2,3]. Hybrid laser–arc welding combines the advantages of using laser light and an electric arc. It is a new welding technology to produce narrow welds with a large penetration depth, which significantly enhances the range of weldable materials. However, while welding high-nitrogen steel, nitrogen dissolves in the steel via the escape of atoms. Because of this, the mechanical properties and corrosion resistance of the weld are significantly lower than those of the base metal, limiting the application of high-nitrogen steel in specific environments. To alleviate this problem, measures that can be taken to improve the corrosion resistance of high-nitrogen steel during welding have become an important topic for experts and scholars in the field [4,5,6,7].

During the welding of high-nitrogen steel, in a high-temperature and low-pressure environment, the nitrogen atoms in steel can freely dissolve during high-pressure melting. These atoms also diffuse toward the direction where the nitrogen content is low. As a result, the nitrogen atoms escape from steel upon the combination of the nitrogen molecules. The stirring action of the laser keyhole on the molten pool accelerates the movement of nitrogen atoms. As the molten pool of the weld cools down, the nitrogen molecules that fail to escape in time accumulate to form nitrogen pores in the weld [8,9,10]. To study the effect of nitrogen loss on the properties of high-nitrogen steel during welding, Li et al. [11] investigated the morphology, microstructure, and microhardness of the zones in a high-nitrogen steel welded joint under different heat inputs during laser–arc welding. The results showed that the weld structure comprises austenite and a small amount of ferrite. With the increase in the heat input, the ferrite content increases, and the ferrite dendritic trunk grows and becomes thicker. Moreover, secondary dendrites are distributed on both sides of the dendritic trunk. Wang et al. [12] studied the influence of laser energy, arc energy, and vibration frequency on the porosity of high-nitrogen steel welds. The results showed that the porosity first increases and then decreases with the increase in the arc or laser energy. Furthermore, an appropriate amount of arc or laser energy can effectively limit the number of pores in the welds. Rui et al. [13] studied the influence of different welding parameters on the electrochemical corrosion properties of a high-nitrogen steel surfacing layer. The corrosion resistance of the surfacing layer first increased and then decreased with the increase in the weld current, laser power, and surfacing speed. Currently, research on the welding of high-nitrogen steel has mainly focused on the mechanical properties of the welded joints upon varying the heat input, welding filler, and shielding gas components. However, research on the corrosion resistance of high-nitrogen steel welded joints has not been extensively conducted.

This study used a hybrid laser–arc welding technology to conduct welding tests on a 6 mm-thick high-nitrogen steel plate at different welding heat inputs. The effect of heat inputs on the corrosion resistance of high-nitrogen steel welded joints was studied to reveal the corrosion resistance and mechanisms of these joints.

## 2. Experimental Materials and Methods

The test material used in this study was high-nitrogen austenitic stainless steel. The filler material was a welding wire made of austenitic stainless steel with a diameter of 1.2 mm, and the welding plate dimensions were 400 mm × 100 mm × 6 mm. Before welding, a “Y” groove was pre-opened with an angle of 30°. The length of the blunt edge was 3 mm, and the butt-welding method was used. Table 1 presents the main chemical composition of the wire and welding plate with a butt gap of 0.4 mm.

**Figure 1:**
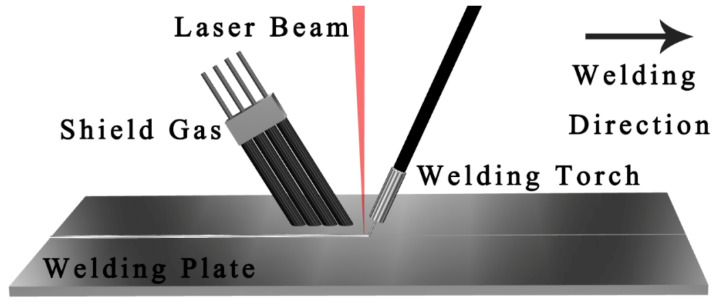
Schematic of the hybrid welding system.

For the welding equipment, we used the H4006D-type Nd: YAG continuous solid laser (TRUMPF, Ditzingen, Germany) and YD-350AG2HGE-type MIG/MAG welder (Panasonic, Osaka, Japan) to form an arc in front of the welding equipment, and a hybrid laser welding system on the rear and side axles. Figure 1 shows the schematic of the hybrid welding system.

Before welding, the burrs and oxide layers near the grooves were removed with sandpaper, and the surface was wiped with acetone to remove the grease and impurities. The vertical welding plate was irradiated by a laser. The angle of the welding gun was 25°, the laser power was in the range of 2–2.8 kW, the defocus was −2 mm, the welding speed was 0.7 m/min, the welding current was 220 A, the welding voltage was 24.8 V, the heat source spacing was 3 mm, and the arc protection gas was 5%N_2_ + 95%Ar supplied at a rate of 18 L/min.

After welding, the excess height of the weld seam was removed with an angle grinder. A disc sample with a diameter of 10 mm was cut using a wire cutter in the middle of the weld on the vertical weld plate surface. According to the data, the excess height of 3 mm for the electrochemical test sample was removed. After the sample was inlayed, the excess height surface was removed with 240–2000-mesh sandpapers, ground, and polished. The surface of the weld seam of the sample was covered with an oil-based resin, whereas the rest was covered with an insulating paint. The oil-based resin was removed after the paint was dry. The sample welds were encapsulated as test areas on an electrochemical test instrument.

We used the ZENNIUM (Zahner, Kronach, Germany) electrochemical workstation as the electrochemical test instrument. A standard three-electrode system was adopted as the electrode: the working electrode was the sample, the auxiliary electrode was the Pt electrode, and the reference electrode was a silver chloride electrode. The electrolyte was a NaCl solution with a mass fraction of 3.5%. The polarization test potential ranged from −2.5 to 2.5 V, and the scanning rate was 1 mV/s. Before the polarization test, an open-circuit test was performed to ensure the stability and accuracy of the test results.

After the electrochemical test, the test specimen was cleaned with alcohol and stored in a vacuum for 24 h. Metallographic microscopy (Leica DM2700M, Weztlar, Germany) and scanning electron microscopy (SEM, JEOL JSM-6510LA, Beijing, China) were used to analyze the weld corrosion surface morphology, dendritic distribution, and microstructural characteristics.

## 3. Experimental Results and Analysis

### 3.1. Influence of Laser Power on Electrochemical Properties

**Figure 2:**
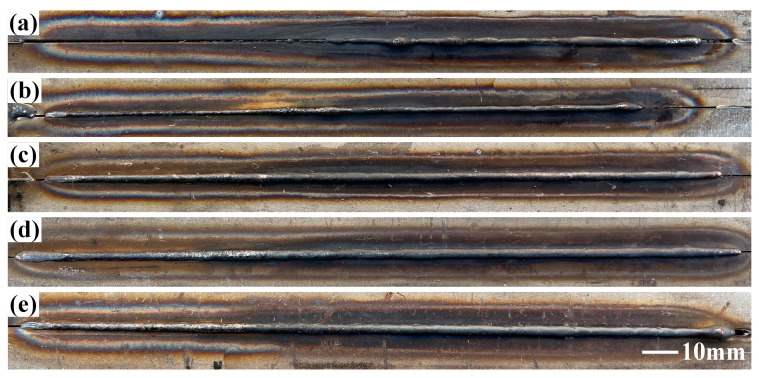
Schematic diagram of weld bottom at (**a**) 2 kW, (**b**) 2.2 kW, (**c**) 2.4 kW, (**d**) 2.6 kW, and (**e**) 2.8 kW.

From the results of the pre-experiment conducted in the early stage, poor weld formation is noted at low laser powers. Figure 2a shows the absence of penetration at the bottom of the weld at a laser power of 2 kW. Meanwhile, Figure 2b shows the penetration at the bottom of the weld with nonuniform weld beads. Figure 2c–e shows excellent weld formation.

**Figure 3:**
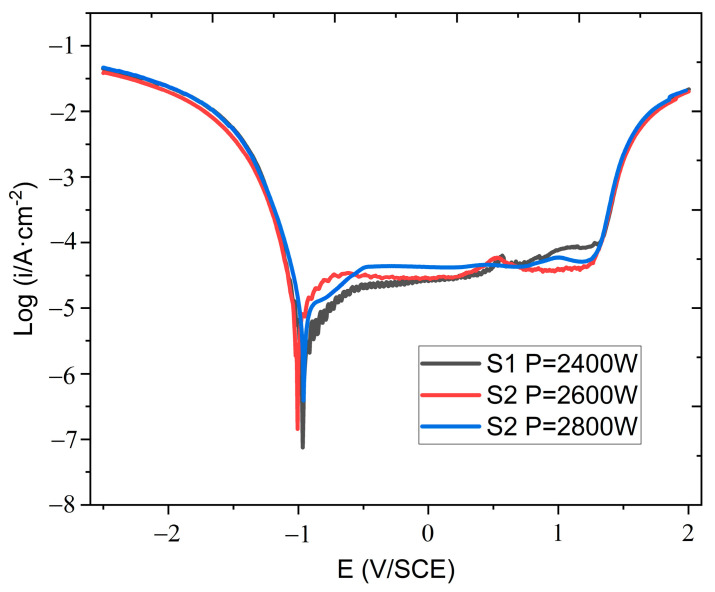
Electrochemical polarization curve.

Therefore, the laser power was set in the range of 2.4–2.8 kW for the subsequent experiments. Three or more electrochemical tests were performed on each sample, and a smoother curve was selected for the fitting analysis. The increase in the laser power increased the weld heat input, as shown in Table 2. Figure 3 shows the polarization curve of the electrochemical test. After entering the stable passivation region, the passivation current density (*I_p_*) of samples S1–S3 increased with the increase in the laser power. *I_p_* was collected at −0.5 V/SCE. Among all the samples, S1 exhibits the best pitting corrosion resistance, whereas S3 has the worst pitting corrosion resistance. The *I_p_* results follow the order S3 > S2 > S1. With the increase in the laser power, the pitting corrosion resistance of the sample surface deteriorated. The polarization curve test parameters were fitted using the Zahner Analysis software by Butler–Volmer. As shown in Table 3, the corrosion current density (*I_c_*) of the sample increases with the increase in the laser power. Moreover, the corrosion rate of the sample gradually increases. This indicates that the corrosion resistance of the weld deteriorated with the increase in the laser power.

### 3.2. Influence of Laser Power on Weld Structure

**Figure 4:**
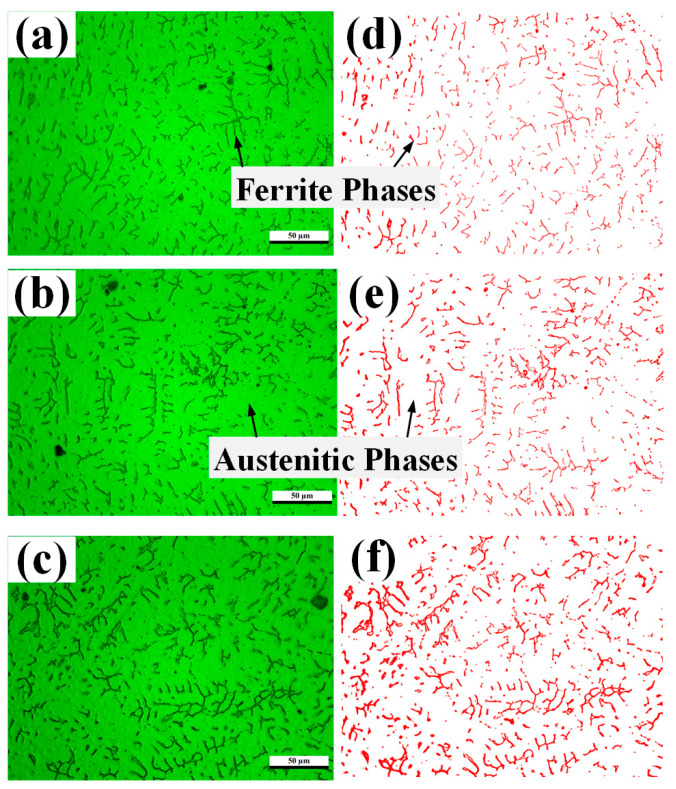
Metallographic structure and ferrite shape of the weld at different powers: (**a**,**d**) 2.4 kW, (**b**,**e**) 2.6 kW, and (**c**,**f**) 2.8 kW.

The results of the electrochemical corrosion test show that the increase in the welding laser power increased the heat input of the welded joint and decreased the corrosion resistance of the weld. This phenomenon can be ascribed to the internal microstructure of the weld, in which the high-nitrogen austenitic stainless steel weld is composed of austenite and ferrite [11]. With the increase in the heat input, the cooling time of the weld pool and growth time of the weld dendrites increase. As shown in Figure 4a, after the electrolytic corrosion of the upper surface of the weld with 10% oxalic acid solution, the weld structure is composed of austenite and ferrite, where the bright and dark areas in the figure are the austenitic and ferritic phase, respectively. At a low laser power, ferrite exhibits a worm-like and small-rod-shaped structure. With the increase in the laser power, the thickness and length of the main stem of the ferrite dendritic arms increase, along with the higher density of their distribution.

**Figure 5:**
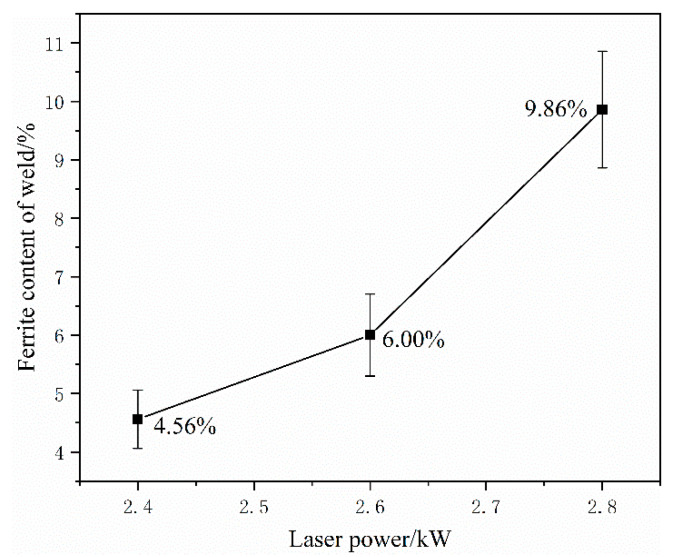
Ferrite dendrite content in the weld.

The proportion of ferrite area in the corrosion plane was calculated using the Image Pro Plus software. The ferrite dendrite content of the corrosion plane of the weld increases with an increase in the heat input. As shown in Figure 5, the ferrite dendrite content is 4.56%, 6.00%, and 9.86% at 2.4 kW, 2.6 kW, and 2.8 kW, respectively. The ferrite dendrite content increases with the heat input to the weld. Combined with the electrochemical corrosion test, the corrosion resistance of the weld positively correlated with the heat input. Therefore, the corrosion resistance of the weld is related to its ferrite dendrite content.

### 3.3. Effect of the Ferrite Content on the Corrosion Resistance of the Welds

With the increase in the laser power, the welding heat input and the time required to cool the weld increased. The weld structure is transformed from the austenite of the base metal to austenite + ferrite. In addition, the ferrite dendrites in the molten pool are elongated for a longer duration. With the increase in the heat input, the ferrite dendrite content gradually increases. The maximum solubility of the C content of austenite is equivalent to the mass fraction ω(C) = 2.11%, whereas that of the ferrite is ω(C) = 0.0218%. This is attributed to the distribution of the C atoms as solvents in the gap between the austenite and ferrite octahedral lattices, thereby forming a gap solid solution, whereas the face-centered cubic lattice of austenite has a wider lattice gap than the body-centered cubic lattice of ferrite [14]. Therefore, during the transition from austenite to ferrite in the molten pool, the C atoms inside the crystal are extruded and accumulated between the austenitic and ferrite phases to form M_23_C_6_.

During solidification, primary carbides are formed at the grain boundaries and M_23_C_6_ precipitates, thereby confirming the formulation of time–temperature–precipitation (TTP) diagrams for the carbon-containing stainless steel. M_23_C_6_ precipitates are not observed in the welding process because of the variability of the precipitation distributions at different cooling rates. The difference in the cooling rate prompted the formation of precipitates with different morphologies and sizes, including fine precipitates, clusters of precipitates, and precipitates that formed lamellar bands. According to the Ni eq and Cr eq values of the alloy, the ferrite content can also be determined from the Schaeffler–Delong diagram. The different properties of the alloy obtained with different cooling rates are unrelated to the ferrite ratio. Thus, the changes in the properties of the alloy are ascribed to the effects of different cooling rates depending on the cooling environment. The finely dispersed carbide precipitates prevent grain coarsening and brittle fracture, which is the case for a low cooling rate during cooling at the lowest laser power. The toughness of the alloy gradually decreased with the increase in the cooling rates [15]. Xin [16] studied stainless steel welded joints with austenite as the matrix and found minimal M_23_C_6_ precipitation in the weld. Therefore, M_23_C_6_ has a negligible effect on the corrosion resistance of high-nitrogen steel welded joints and is not discussed herein.

**Figure 6:**
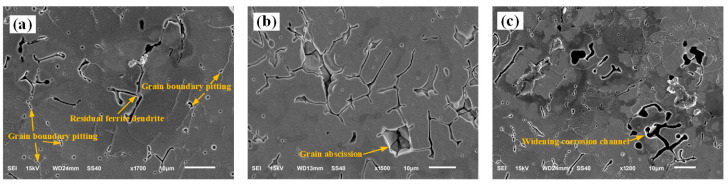
Corrosion morphology of the weld under different laser powers: (**a**) 2.4 kW, (**b**) 2.6 kW, and (**c**) 2.8 kW.

**Figure 7:**
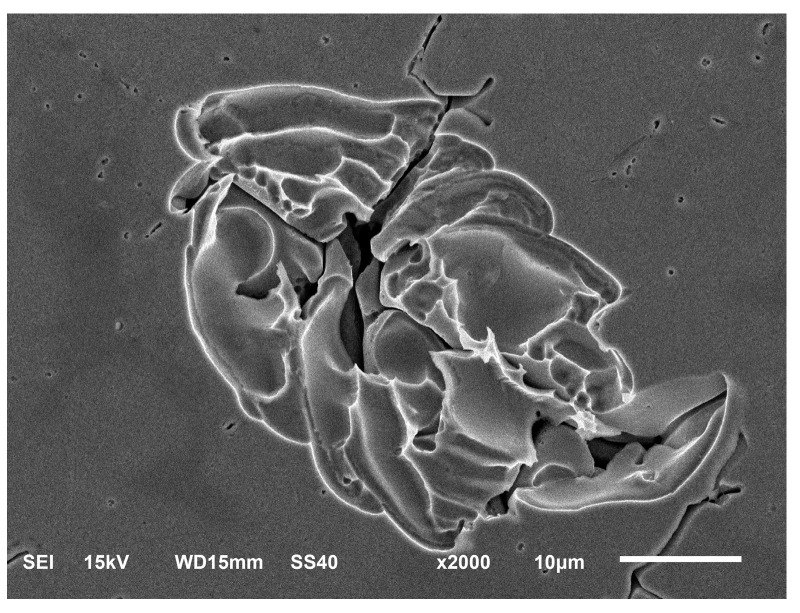
Corrosion morphology of ferrite dendrites after dissolution.

An SEM observation of the sample surface after galvanic corrosion shows that corrosion first occurred at the interface between the austenite and ferrite, forming corrosion pits on the side of the ferrite phase, as shown in Figure 6a. Subsequently, Cl^−^ in the corrosive medium enters the interface between the two phases, thereby accelerating the corrosion process. During the electrochemical anodic reaction, local galvanic corrosion occurred on the austenite and ferrite phases. The ferrite phase is first corroded as the anode in the galvanic pair. As the corrosion progresses, the ferrite dendrites gradually dissolve by corrosion from the two-phase interface, the volume decreases, the number of corrosion channels gradually increases along the dendrite growth direction [17], and the austenite phase is protected as a cathode and is not corroded, as shown in Figure 6b. After the ferrite dendrites are completely dissolved or detached from the corrosion channels, only the austenite phase and corrosion channels remained. When all the grain boundaries around the austenite grains are corroded and only the corrosion channel is left, the austenite grain falls off from the center of the weld, as shown in Figure 6b. Figure 6c shows the continuous reaction of the corrosive medium with the weld specimen, beginning of the corrosion and dissolution of the austenite phase, and larger size and width of the corrosion channels than those of the ferrite dendrites. Pitting corrosion occurs along the corrosion channels on the austenite crystals, thereby forming corrosion pits and promoting the dissolution of austenite by corrosion, as shown in Figure 7.

The increase in the heat input due to the laser power increases the content of the ferrite dendrites at the center of the weld. In the electrochemical corrosion process, with the increase in the laser power, the corrosion current and rate of the weld specimens exhibit an increasing trend. This indicates that the change in the ferrite dendrite content is the main factor for the reduction of the weld corrosion resistance. The increase in the ferrite content increases the number of the ferrite and austenite interfaces and promotes corrosion. Furthermore, with the increase in the number of the ferrite dendrites, the contact area of the reactants during galvanic corrosion increases, the reaction speed is accelerated, and the ferrite dendrites leave more corrosion channels after complete corrosion and dissolution. Thus, the austenite grains fall off, the weld interior becomes looser, more surfaces are exposed to the corrosive medium, and the corrosion resistance of the weld decreases.

### 3.4. Simulation Using First-Principles Calculations

**Figure 8:**
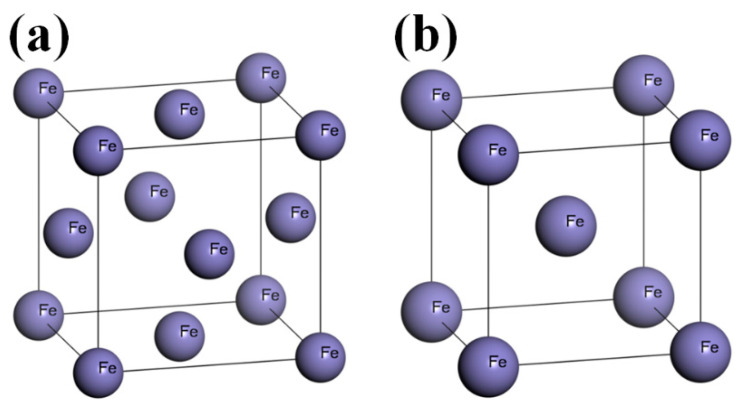
First-principles calculation model: (**a**) Fcc-Fe and (**b**) Bcc-Fe.

**Figure 9:**
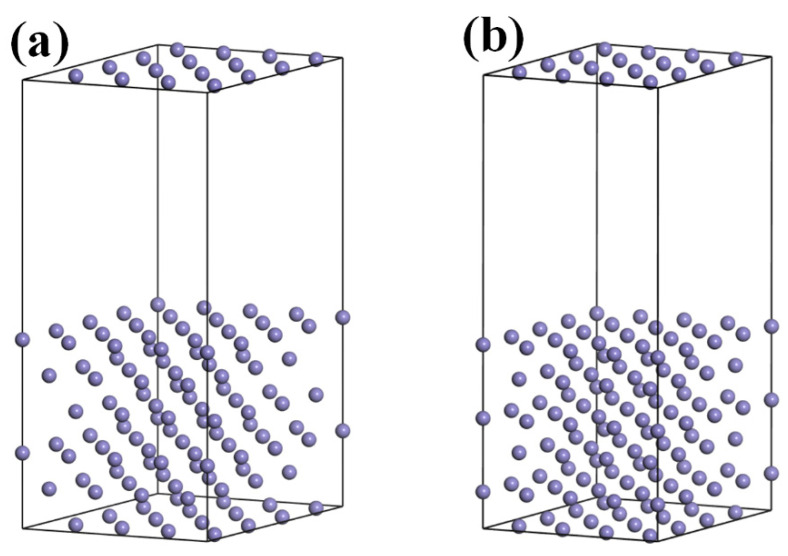
First-principles calculation surface model: (**a**)Fcc-Fe (111) and (**b**)Bcc-Fe (110).

**Figure 10:**
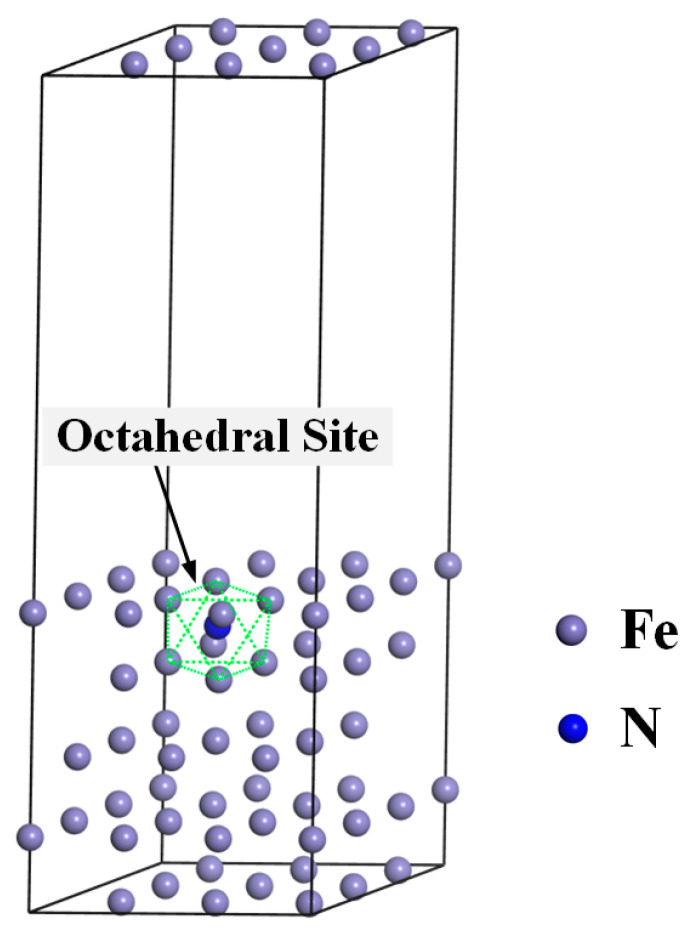
Fcc-Fe (111)-N model calculation surface model.

The metallographic structure of the high-nitrogen steel weld studied herein comprised austenitic and ferrite phases. The unit cell energy of the face-centered cubic and body-centered cubic units and the energy of the austenitic dense arrangement (111) and ferritic-dense arrangement (110) surfaces were calculated using the first-principles calculation software Materials Studio, as shown in Figure 8 and Figure 9. Figure 10 shows the surface calculation model of the high-nitrogen steel. The nitrogen atoms are solidly dissolved in the octahedral gap of the second layer of atoms. The calculation uses the generalized gradient approximation to exchange the correlation energy and the Perdew–Burke–Ernzerhof function. The geometric structure optimization energy converges to 1.0 × 10^−6^ eV/atom, with the maximum force convergence of 0.01 eV/Å, maximum stress convergence of 0.02 GPa, maximum displacement convergence of 0.001 Å, and electron self-consistent cycle error of 1.0 × 10 ^−6^ eV/atom. The pseudopotential was obtained using Ultrasoft pseudopotential. To reduce the impact of the lattice periodicity on the calculation, the surface model was subjected to a 3 × 3 × 1 supercell treatment. A vacuum layer with a length of 15 Å was added to the upper layer of the surface to reduce the interaction between different layers of atoms in the Z-direction. The coordinates of the lower three layers of the atoms in the six-layer atomic surface structure were fixed. In the subsequent surface relaxation process, the cell optimization was set to none, that is, the lattice was not optimized. The default lattice constant of the bulk model was the surface lattice constant. The *k* point of the crystal cell calculation was selected as 8 × 8 × 8. The cutoff energy of the crystal cell calculation was selected as 500 eV. The *k* point of the crystal plane calculation was selected as 3 × 3 × 1. The cutoff energy of the crystal plane calculation was selected as 400 eV. All calculations considered the spin polarization.

When cutting a bulk material into surfaces, surface energy is required to destroy internal interatomic forces. The lower the surface energy, the more stable the surface structure is. Therefore, surface energy is an essential criterion for evaluating surface stability. The surface energy calculation formula is shown as follows:

(1) $$E_{surface}=\left[E_{slab}-\left(\frac{N_{slab}}{N_{bulk}}\right)\cdot E_{bulk}\right]/2A$$

where *E_surface_* is the surface energy, *E_slab_* is the surface structure energy after the structural optimization, *N_slab_* is the number of atoms in the surface mechanism, *N_bulk_* is the number of atoms in the bulk cell, *E_bulk_* is the energy after the bulk structural optimization, and *A* is the surface area of the surface structure.

The electron work function is used to characterize the minimum energy required to move an electron from the interior of a metal material to infinity in a vacuum. The larger the electron work function, the stronger the binding ability of the metal to the electron. The calculation formula for the electron work function is

(2) $$\phi =E_{vacuum}-E_{fermi}$$

where ϕ is the electron work function, *E_vacuum_* is the energy level of the vacuum layer, and *E_fermi_* is the Fermi energy level.

**Figure 11:**
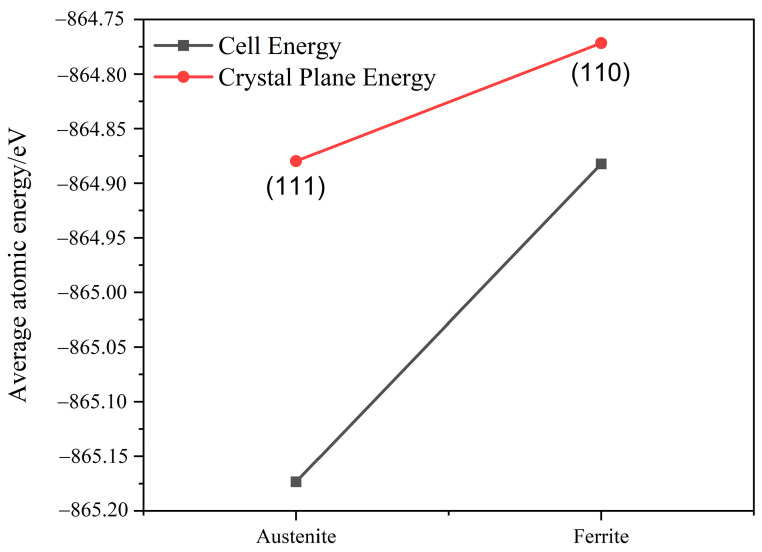
Cell and crystal surface energy.

Using the same calculation accuracy, the average atomic energy in the austenite crystal cell was −865.17 eV, which is greater than the absolute value of the average atomic energy in the ferrite crystal cell (−864.88 eV). Additionally, the average atomic energy in the austenite (111) plane structure was −864.87 eV, which is greater than the absolute value of the average electronic energy in the ferrite (110) plane structure (−864.77 eV). This indicated that the face-centered cubic unit cell structure is more stable than the body-centered cubic unit cell structure. Moreover, the austenite (111) plane has better structural stability than the ferrite (110) surface, as shown in Figure 11. The calculations show that the electron work function of the austenite (111) surface is 4.72 eV, which is greater than that of the ferrite (110) surface (4.60 eV). Comparison with data in previous studies revealed that the errors obtained in this work were within reasonable limits, thereby confirming the reliability of this calculation. The error may be ascribed to the difference in the calculation accuracy. The electron work function of the austenite surface structure with a solid solution of nitrogen atoms is 4.80 eV, which is greater than that of the non-solid solution surface.

As shown in Table 4, the magnitude of the electron work function can reflect the difficulty of the surface atoms to gain or lose electrons, i.e., a higher electron work function indicates higher energy required for the electrons to escape the surface atom. The surface energy of the austenite (111) surface was 2.45 J/m^2^, which was lower than that of the ferrite (110) surface (2.77 J/m^2^). The surface energy can also reflect the stability of the surface structure, i.e., a lower surface energy indicates a more stable surface structure. The surface energy of the austenite surface structure with solid-solution nitrogen atoms is 2.28 J/m^2^, which is lower than that of the non-solid solution surface.

These results support the corrosion sequence of the ferritic and austenitic phases in the galvanic corrosion tests. As the ferrite content of the weld increases, the corrosion resistance of the weld decreases. The first-principles calculation results show the lower structural stability of ferrite than that of austenite. The surface structure of austenite has higher stability after the addition of solid-solution nitrogen atoms.

## 4. Discussion

During the welding process, an increase in the laser power causes a decreasing trend in the corrosion resistance of the weld. Meanwhile, the ferrite dendrite content of the weld tissue increased with increasing laser power, as observed using metallographic microscopy. In the electrochemical experiments, the welds with high ferrite dendrite content exhibit high corrosion currents. During the corrosion process of the weld, ferrite is preferentially dissolved and corroded. After the ferrite phase is dissolved and corroded, the austenite phase starts to corrode and dissolve, as observed using SEM.

According to previous research [20,21,22], galvanic corrosion occurs when metals with different potentials come into contact with each other in a corrosive medium. In this study, the ferrite phase in the weld is in contact with the precipitated austenite phase, resulting in the galvanic corrosion, as observed using SEM. Hou et al. [19] revealed the mechanism of steel-induced galvanic couple corrosion in typical inclusions by studying the relationship between the electron work function of Al–Ti–Mg steel and galvanic corrosion. During the galvanic corrosion of body phases with different electron work functions, the body phase with a lower energy work function is preferentially dissolved and corroded. Zhibo [18] studied the electron work function of different crystal planes of each phase in stainless steel and found that as the work function increased, the ability of the material surface to lose electrons and participate in the reaction as a cathode during galvanic corrosion decreased. In contrast, when the phase has a lower electron work function, it is the first to be corroded as the anode during galvanic corrosion. The first-principles calculation results in this study show that ferrite in the weld is first corroded as the anode and austenite is protected as the cathode. As the ferrite content increases, the corrosion rate of the weld increases, which explains the different factors inducing weld corrosion resistance owing to the changes in the welding heat input. Bo [7] noted the decrease in the nitrogen content of the weld after welding high-nitrogen steels upon the variation of the degrees of nitrogen loss, which decreased the corrosion resistance of the weld. The work function and surface energy of the face-centered cubic (111) surface structure of the solid-solution nitrogen atoms with nitrogen content similar to that of the base material are higher than those of the nitrogen-free structure. The escape of the nitrogen atoms from the base material reduced the stability of the structure and enhanced the corrosion tendency. Therefore, the root cause of the deterioration of the corrosion resistance of high-nitrogen steels is the decrease in the nitrogen content and increase in the ferrite content of the weld at a high heat input.

## 5. Conclusions

The corrosion resistance of the weld specimens varied when subjected to welding at different laser powers. As the laser power was increased, the corrosion resistance decreased, the *I_p_* increased in the stable passivation region, and the *I_c_* and corrosion rate showed an upward trend.

The weld structure under different laser powers comprised austenite and ferrite phases. With an increase in laser power, the ferrite content gradually transformed from fine strips into dendrites. With a further increase in laser power, the dendrite arms gradually grew, thereby forming secondary dendrite arms. The ferrite dendrite content increased from 4.56% at 2.4 kW to 9.86% at 2.8 kW.

Increasing the ferrite content of the weld reduced the corrosion resistance of the high-nitrogen steel welded joints. Corrosion started at the interface between the austenite and ferrite phases, which formed corrosion pits, followed by the invasion of the corrosion medium. Ferrite was dissolved to form corrosion channels. After the ferrite phase was completely corroded and dissolved, the austenite phase began to corrode and dissolve.

The absolute value of the average atomic energy of the cell and surface structure of ferrite was lower than that of austenite. The lower surface energy of austenite indicates its higher work function than that of ferrite.

## Figures and Tables

**Table 1:** Main chemical composition (wt.%) of the base metal and welding wire.

Element	C	Cr	Mn	Ni	Mo	Si	N
Base Metals	0.148	20.07	16.00	0.47	—	0.49	0.56
Welding Wire	0.09	21.00	1.6	9.00	0.37	—	—

**Table 2:** Heat input parameters for the hybrid weld.

Group	Current/A	Voltage/V	Laser Power/kW	Linear Energy/(J/cm)
S1	220	24.8	2.4	6733.71
S2	220	24.8	2.6	6905.14
S3	220	24.8	2.8	7076.57

**Table 3:** Experimental parameters of the polarization curve.

Group	Corrosion Potential *E*_corr_/V	Corrosion Current Density *I*_corr_/μA·cm^−2^	Corrosion Rate *v*_c_/mm·year^−1^
S1	−0.9671	9.86	115 × 10^−3^
S2	−1.0074	22.9	267 × 10^−3^
S3	−0.9609	27.2	316 × 10^−3^

**Table 4:** Different surface work functions and surface energy.

Surface	Work Function/eV	Ref.	Surface Energy/(J·m^−2^)	Ref.
Austenitic-N-(111)	4.80		2.28	
Austenitic (111)	4.72	5.05 [18]	2.45	2.67 [18]
Ferrite (110)	4.60	4.50 [19]	2.77	2.65 [19]

## Data Availability

The data is available upon request from corresponding author.
